# The hydrodynamic regime drives flow reversals in suction-feeding larval fishes during early ontogeny

**DOI:** 10.1242/jeb.214734

**Published:** 2020-11-05

**Authors:** Krishnamoorthy Krishnan, Asif Shahriar Nafi, Roi Gurka, Roi Holzman

**Affiliations:** 1 School of Coastal and Marine Systems Science, Coastal Carolina University, Conway, SC 29526, USA; 2 School of Zoology, Faculty of Life Sciences, Tel Aviv University, Tel Aviv 69978, Israel; 3 The Inter-University Institute for Marine Sciences, PO Box 469, Eilat 88103, Israel

**Keywords:** *Sparus aurata*, Reynolds numbers, Feeding kinematics

## Abstract

Fish larvae are the smallest self-sustaining vertebrates. As such, they face multiple challenges that stem from their minute size, and from the hydrodynamic regime in which they dwell. This regime, of intermediate Reynolds numbers, was shown to affect the swimming of larval fish and impede their ability to capture prey. Prey capture is impeded because smaller larvae produce weaker suction flows, exerting weaker forces on the prey. Previous observations on feeding larvae also showed prey exiting the mouth after initially entering it (hereafter ‘in-and-out’), although the mechanism causing such failures had been unclear. In this study, we used numerical simulations to investigate the hydrodynamic mechanisms responsible for the failure to feed caused by this in-and-out prey movement. Detailed kinematics of the expanding mouth during prey capture by larval *Sparus aurata* were used to parameterize age-specific numerical models of the flows inside the mouth. These models revealed that for small larvae which expand their mouth slowly, fluid entering the mouth cavity is expelled through the mouth before it is closed, resulting in flow reversal at the orifice. This relative efflux of water through the mouth was >8% of the influx through the mouth for younger ages. However, similar effluxes were found when we simulated slow strikes by larger fish. The simulations can explain the observations of larval fish failing to feed because of the in-and-out movement of the prey. These results further highlight the importance of transporting the prey from the gape deeper into the mouth cavity in determining suction-feeding success.

## Abstract (precis)

**Highlighted Article:** Larval fish struggle to capture their prey, which sometimes slips out of the mouth as it is closing. A computational fluid dynamics (CFD) model reveals that this is due to reversal of the flows within the mouth, which is characteristic of slow strikes by small larvae.

## INTRODUCTION

Most marine fish reproduce by broadcasting small eggs (∼1 mm in diameter) into the open ocean, providing no parental care to the hatching larvae (Blaxter, 1988; Cowen, 2002; Houde, 1987). Typically, larvae deplete their yolk sac after a few days (usually 3–7 days, depending on the species, temperature and environmental conditions) and must feed autonomously in order to gain the necessary resources to complete their development (Blaxter, 1988; Cowen, 2002; Houde, 1987). Despite an immense variation in adult body sizes and life-history strategies, the tiny eggs and larvae are nearly ubiquitous across marine fish species, as is the lack of parental care (Barneche et al., 2018). Fish larvae that hatch at sizes as small as 2–3 mm are thus the smallest self-sustaining vertebrates. In almost all documented examples of larval fish feeding, prey capture is accomplished using ‘suction feeding’, a characteristic behavior in which fish sequentially open their mouth, expand their buccal cavity, and open the gill covers to generate a unidirectional flow of water that carries their prey into the mouth (Blaxter, 1980; Day et al., 2015; Drost et al., 1988; Holzman et al., 2015).

In the wild, larval fish suffer dramatic mortality rates (Hjort, 1914; Houde, 1987). It is estimated that >90% of the brood is eradicated during the ‘critical period’, extending from the time of first feeding until the larva is ready to settle in its juvenile habitat. During this period, larval fish undergo dramatic morphological and developmental changes, including ossification of the cranium and vertebrae, degradation of the fin fold and the development of fin rays, as well as the continuous growth and development of the eyes (Blaxter, 1988; Kavanagh and Alford, 2003). Concomitantly, coordination of feeding and swimming motions and motor pattern change and improve (Westphal and O'Malley, 2013). The physical growth of the larvae, coupled with the development of stronger muscles that support faster movements, leads to an ontogenetic transition in the ways in which larvae interact with their fluid environment (China and Holzman, 2014; Holzman et al., 2015). Being small and slow, young larvae dwell in a domain of intermediate Reynolds numbers (1<*Re*<100), in which the viscous forces are non-negligible compared with the inertial ones. This hydrodynamic regime has been shown to impede the feeding rates of larval fishes. For example, 8 days post-hatching (dph) *Sparus aurata* larvae, whose suction flows were characterized by *Re*<10, failed to capture non-evasive prey in ∼80% of their feeding strikes (China and Holzman, 2014; China et al., 2017). Feeding experiments in seawater treated with dextran to enhance its viscosity revealed that the feeding rates of larvae were determined primarily by the hydrodynamic environment, described by the Reynolds numbers that characterized the feeding events (China and Holzman, 2014; Holzman et al., 2015), and were independent of larval development. This was indicated by the observation that larger larvae (13 and 23 dph) that were feeding in dextran-treated seawater displayed feeding rates equivalent to those of the 8 dph larvae in unmanipulated water. Larvae that were raised in media with increased viscosity compared with seawater expressed elevated levels of hunger-related neuropeptides (Koch et al., 2018) and suffered higher mortality rates (Yavno and Holzman, 2018). Furthermore, the probability of executing successful prey-acquisition strikes increased with increasing *Re* (China et al., 2017). Transition into higher *Re* also improves larval ability to capture highly evasive prey such as copepods (Jackson and Lenz, 2016; Sommerfeld and Holzman, 2019; Yaniv et al., 2014).

Observations using high-speed videos indicate that one of the reasons for failure in prey-acquisition strikes is the occurrence of ‘in-and-out’ events, in which the prey is carried into the mouth by the suction flow, but is then expelled while the mouth is closing (China et al., 2017; Holzman et al., 2015). The suction flows in these in-and-out events were characterized by lower *Re* compared with those in successful events. Furthermore, in-and-out strikes were initiated from a greater distance to the prey and were slower (had a longer time to peak gape) compared with unsuccessful events in which the prey did not even enter the mouth (China et al., 2017). A flow visualization study revealed flow reversals in larval zebrafish, where fluid was expelled from the cavity through the mouth during its closure. These flow reversals occurred in 3.5–4 mm long larvae at the time when the mouth started closing (Pekkan et al., 2016). This finding is in sharp contrast to those from adult fish, where flow reversals are rare and minor (Jacobs and Holzman, 2018). However, the extent of these flow reversals across species and developmental stages is unclear, as are as the hydrodynamic conditions under which they occur.

Here, we used computational fluid dynamics (CFD) to investigate the fluid dynamics of suction-feeding larval fish. Following Yaniv et al. (2014), we constructed a model of an expanding buccal cavity, incorporating an anterior-to-posterior wave of buccal expansion over time (Bishop et al., 2008). Our modeling improves previous attempts to model the buccal cavity in larval fish by including the opening of the gill covers at the posterior end of the mouth, a hallmark feature of suction-feeding in fishes (Lauder, 1985; Westneat, 2005). The opening of the gills enables the generation of unidirectional flows into the mouth while the gape is closing (van Wassenbergh, 2015). The model was parametrized based on observed strike kinematics of *Sparus aurata* larvae, ranging in age from first feeding to metamorphosis. Using these kinematics, we quantified the flow speeds, and the influx and efflux into the mouth and out of the gills for six larval ages. We then characterized the extent of flow reversals, the flow conditions in which they occur, and the role of hydrodynamics and kinematics (behavior) in driving these flow reversals.

## MATERIALS AND METHODS

### Study organisms

We re-analyzed high-speed videos of suction-feeding gilthead sea-bream larvae (*Sparus aurata* Linnaeus 1758) feeding on rotifers (*Brachionus rotundiformis*; ∼0.16 mm in length) from previously published datasets (China and Holzman, 2014; China et al., 2017). *Sparus aurata* is a pelagic spawner, hatching at ∼3.5 mm. Feeding initiates at ∼5 dph at a body length of ∼4 mm. Larvae reach the stage of flexion at ∼21–24 dph, at a length of 7–10 mm, and recruit at 34–35 dph; however, the timing of these life-history transitions depends on temperature, feeding regime and other environmental conditions. The prey, *B. rotundiformis*, is a species of planktonic rotifer, actively swimming at ∼0.2 mm s^−1^, an order of magnitude slower than the swimming speed of the larvae, and their escape response is considered weak (China and Holzman, 2014; China et al., 2017). Rotifers are universally used as the standard first-feeding food in the mariculture industry.

### High-speed videos

Suction-feeding events of larval fish were recorded using high-speed video (500 and 1000 frames s^−1^) as described previously (China and Holzman, 2014; China et al., 2017). In these experiments, fish swam freely in an aquarium, and their orientation with respect to the camera included lateral, dorsal and ventral views. From the larger dataset of prey acquisition strikes, we selected 63 clips in which we could clearly track the kinematics of mouth opening as well as either the hyoid (using the lateral view of the fish) or the gill covers (using dorsal or ventral views) throughout the prey acquisition strikes. Clips were selected for fish at the ages of 8–9, 12–13, 17–18, 22–25, 30 and 35–37 dph (hereafter 8, 13, 18, 23, 30 and 37 dph; *N*=4–14 clips per age group) irrespective of whether the strikes ended in prey capture or not. We used clips that featured different individual larvae (no individual was sampled twice). From videos taken in lateral view, we measured the time of mouth opening and closing, maximal mouth diameter, the time of initiation, peak hyoid displacement and its maximal excursion, and the time of gill cover opening and closing (when clearly visible). From videos taken in either the dorsal or ventral view, we measured the time of mouth opening and closing, the time of initiation, and peak gill cover displacement and its maximal excursion, and the corresponding parameters at the base of the gill covers (first gill arch). We estimated buccal area as a circle with a diameter equivalent to peak gape diameter and gill area as half the area of a ring with internal and external diameters of the minimal and maximal width (respectively) of the larvae at the distal end of the gill covers. To enable comparisons between different ages and strikes, we express the times of hyoid and gill cover excursions in units of time to peak gape opening (TTPG) in each clip (e.g. dividing the times of peak hyoid depression by TTPG within that strike; Jacobs and Holzman, 2018), and their excursions by peak gape. Not all the parameters were visible in all the clips, resulting in a sparse matrix that was ∼60% full. For example, we were able to reliably measure both gape diameter and buccal length for 22 fish, and both gape diameter and maximal gill cover excursion for 29 fish. We averaged the timing and excursion parameters for each landmark, regressed them against larval age and used the predicted values from the regression to generate characteristic kinematics for each age (Table 1).

**Table 1.**
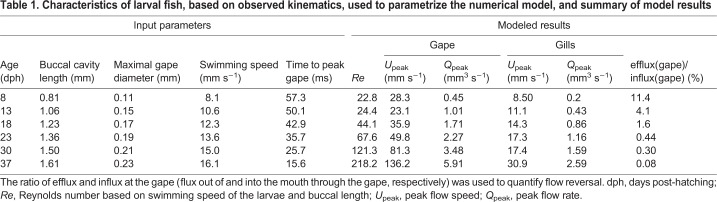
**Characteristics of larval fish, based on observed kinematics, used to parametrize the numerical model, and summary of model results**

### Geometry of the modeled buccal cavity

**Fig. 1.**
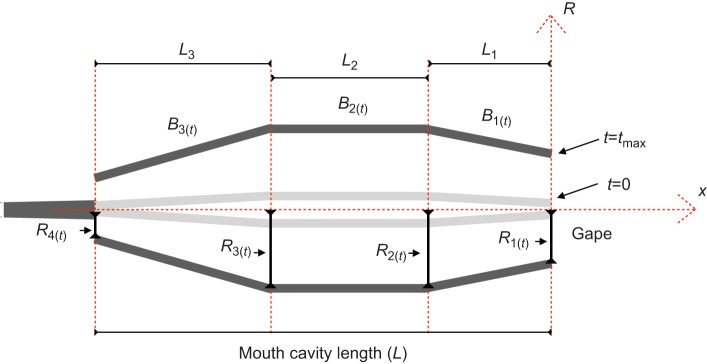
**Schematic description of the model geometry.** Solid black bars indicate the location of the buccal walls under maximal expansion (*t*=*t*_max_); gray bars show the buccal walls before the onset of expansion (*t=*0). The mouth is modeled as three attached cones that expand sequentially (Eqn 1; Fig. 2). *L*_1_–*L*_3_ correspond to the length of the three cones, *B*_1_–*B*_3_ are the lateral surfaces of the three cones, and *R*_1(*t*)_–*R*_4(*t*)_ are the time-dependent radii of the cones. *R*_1(*t*)_ is the gape. The time-dependent increase in *R*_4(*t*)_ represents the opening of the gill slits.

We built our model upon a previous model of mouth cavity expansion suggested by Bishop et al. (2008) and Yaniv et al. (2014), and added cavity contraction and the opening and closing of the opercular slits, a hallmark of suction feeding across fishes (Van Wassenbergh, 2015). In brief, the model comprised three compartments of constant axial length, *L*_1_, *L*_2_ and *L*_3_ (Fig. 1). These compartments represented the region from the mouth opening to the anterior hyoid (*L*_1_), the region spanning the anterior to posterior length of the hyoid (*L*_2_), and the region posterior to the hyoid extending to the opening of the esophagus (*L*_3_). Mouth cavity expansion and contraction was simulated as time-dependent changes in the radii (*R*_1_, *R*_2_, *R*_3_ and *R*_4_) of the bases of the compartments, parametrized according to the observed kinematics of the corresponding landmarks in our larvae (see above; Table 1). The radius *R*_1_ represents the radius of the gape. The lengths *B*_1_, *B*_2_ and *B*_3_ of the surfaces of each compartment varied with time to fit the length variations of the radii *R*_1_, *R*_2_, *R*_3_ and *R*_4_. We simulated mouth expansion for six larval ages (8, 13, 18, 23, 30 and 37 dph) with increasing gape diameter and mouth lengths. Following the measurements reported by Yaniv et al. (2014), internal dimensions of *L*_1_, *L*_2_ and *L*_3_ were 25%, 30% and 45% of the total mouth cavity length *L*, and mouth radii before mouth expansion was set to 2.5% for *R*_1_ and *R*_4_, and 5% for *R*_2_ and *R*_3_.

**Fig. 2.**
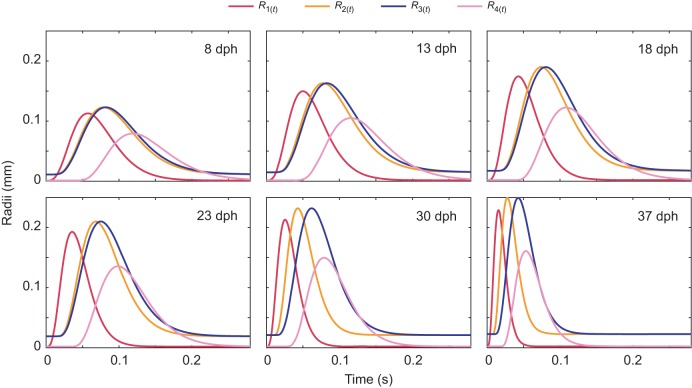
**Buccal expansion kinematics across *Sparus aurata* ontogeny.** Plots depict the time-dependent radii of *R*_1(*t*)_–*R*_4(*t*)_ for 8, 13, 18, 23, 30 and 37 days post-hatching (dph) larvae. Note that the timing and peak radius for each one of the mouth sections *R_i_*_(*t*_max_)_ changes through larval growth. As larvae grow, the overall time to complete the movement generally decreases, whereas the radii (and correspondingly buccal volume) increase.

The pattern of mouth opening was simulated by varying the radii *R_i_*_(*t*)_ of each mouth section (*i=*1:4) with time (*t*) using the following time-dependent exponential function (Eqn 1; modified from Müller et al., 1982):(1)
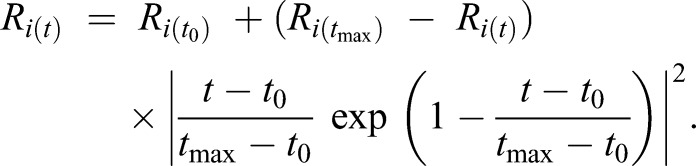
Here, *t*_0_ is *t*=0 and *t*_max_ is the time when the value of *R_i_* is maximal. Note that the radius of each mouth section can have different *R_i_*_(*t*)_, *R_i_*_(*t*_max_)_ and *t*_max_ values according to the mean observed scaling of morphology and kinematics (Table 1, Fig. 2).

Feeding strikes of larval fish of the same cohort are characterized by considerable variation in strike kinematics, including variation in the realized mouth diameter and TTPG (China et al., 2017). Among older (larger) larvae, this variation also encompasses ‘low effort strikes’ wherein older larvae expand their mouth to a diameter smaller than the maximum, using slower kinematics that resembles the kinematics of much younger larvae. Often, such kinematics are associated with failure to capture their prey (China et al., 2017). To investigate the effect of gape kinematics on the flow dynamics within the buccal cavity, we ran numerical simulations (see below) for each of the 23, 30 and 37 dph cases using the observed geometry but with the expansion kinematics and the relative timing of the 8 dph case (time to peak gape *R*_1(*t*_max_)_ of 57.3 ms, and time to peak *R_i_*_(*t*_max_)_ of 70.3, 73.1 and 78.8 ms for sections 2–4, respectively, for all three models; Table 1).

### Computational approach

To simulate the fluid dynamics within the buccal cavity and characterize the flow moving in and out of the mouth cavity, a simplified model of an axi-symmetrical mouth cavity was designed. The boundaries of the mouth cavity in the simulations present a simplified structure featuring a cylindrical wall surrounding the cavity and open inlet and outlet edges at the right and left ends, respectively. The cylindrical wall sections are composed of three length sections that are flexibly connected, and their individual movement is described by the prescribed kinematics (Eqn 1). To represent the body of the fish and supplement the function of the gills, a streamlined elongated body with a length similar to mouth length was designed downstream of the buccal cavity. The body had a small protruding part inside the cavity outlet, with a small (∼10^−3^ mm) gap from the buccal walls at *t*=0. At *t*>0, the mouth started expanding, drawing the fluid in through the gape, followed by the opening of the gap (the gills) based on the prescribed kinematics for *R*_4_. The geometry and the radial expansion of the buccal cavity represent a simplification of the real cavity which can expand differently in the lateral and sagittal planes. Furthermore, we lack data on the internal kinematics and morphology of the cavity, specifically regarding the dimensions of the gill structures and the opercular openings, for which a radially symmetric structure is not realistic. However, our goal was not to model the animal as realistically as possible, but rather to gain an understanding of the basic mechanical principles involved. The validation of the modeled flow speed using data on prey velocity (see Results) indicated that these assumptions are acceptable, but it should be noted that the results should be interpreted in a comparative framework, i.e. used to understand how suction-feeding dynamics changes across early life stages.

The mouth cavity was immersed in a fluid-filled cylindrical domain and placed in the center of the domain. The cylindrical fluid domain has three boundaries: inlet at the right base (face), outlet at the left base (face) and the cylinder's side wall, such that uniform flow was formed to move in the domain along its axis. Water at standard atmospheric conditions was used as the fluid material in the domain. A velocity inlet boundary condition was used at the inlet, with water flowing at 13 mm s^−1^. A pressure-outlet boundary condition with standard atmospheric pressure was set at the domain outlet. The cylinder's side wall of the fluid domain, as well as the walls of the mouth cavity, were represented by a no-slip boundary condition. As the two openings (mouth and gills) of the mouth cavity were left open, it can be expected that the fluid would flow in and out of the mouth cavity naturally depending on its kinematics. To ensure that the domain size did not interfere with the flow inside and around the mouth cavity, the domain had sufficiently large dimensions: approximately 30 times the mouth cavity length along the *x*-direction (flow direction; Fig. 1) and 80 times the peak mouth opening radius along the *R*-direction for each dph case.

The flow field due to expansion of the mouth cavity model was governed by the continuity and momentum conservation equations for incompressible viscous laminar fluid flow in the absence of body force (Ferziger and Peric, 2001). General governing equations for an unsteady, incompressible, viscous laminar flow are:(2)
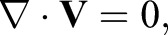
(3)
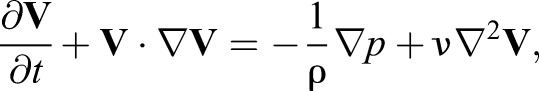
where ρ is the fluid density, **V** is the velocity vector, *p* is the pressure and ν is the kinematic viscosity of the fluid. The flow governing equations were solved using the finite volume-based commercial software package ANSYS fluent (ANSYS, Canonsburg, PA, USA; https://www.ansys.com/products/fluids). The mouth cavity model was designed and meshed using ANSYS workbench. An unstructured (triangular shape) mesh method was chosen to discretize the domain, the cavity and its boundaries. Relatively finer meshes were used inside and around the mouth cavity while coarser meshes were used in the domain distanced from the cavity. To simulate the expansion of the mouth cavity, the dynamic mesh method was utilized. Dynamic meshing corresponds to changing the mesh geometry over time and space based on the prescribed kinematics of the cavity. The kinematic motion of the mouth cavity was prescribed within the fluent solver using the user-defined function ‘DEFINE_GRID_MOTION’ (ANSYS fluent; van Wassenbergh, 2015). This procedure was performed using a user-defined function that was compiled and assigned to each length section of the mouth cavity. The local cell re-meshing method was chosen to re-mesh the grids every two time steps, based on the minimum and maximum cell length and maximum skewness parameters of each cell. To solve the flow equations, a SIMPLE scheme (ANSYS fluent) was employed to carry out the pressure–velocity calculations. Spatial discretization was assigned with a second-order least-square cell-based gradient method while a first-order implicit method was used for time discretization. The complete numerical solution was obtained by ensuring that the convergence criteria of 10^−4^ for the continuity and the flow speed components were attained (ANSYS fluent). Before proceeding with the final simulations, a mesh convergence study was carried out to confirm that a stable solution had been achieved and that the mesh did not influence the solution. For instance, for the 8 dph case, we built three different meshes, with approximately 90,000 cells, 140,000 cells and 300,000 cells. Mesh validation was performed by comparing peak flow speed at both the inlet and outlet for each mesh case, resulting in less than 1% variation between mesh 2 and 3. The mesh with 140,000 cells was therefore chosen for further simulations. Irrespectively, for all the dph cases, the movement of the mouth cavity was simulated for 280 ms with 2800 time steps (each 10^−4^ s).

Flow speed at the mouth and gills (the inlet and outlet of the mouth cavity, respectively) at each time step was calculated as the average of flow speeds across it. Correspondingly, peak flow speed was the flow speed at the time of maximal mouth opening. Flow rates were calculated using an integration of the velocity profile over the mouth and gills (inlet or outlet). In order to resolve the unsteady motion and its coupling effect with the flow field, our simulations were performed using an unstructured grid that corresponded to the changing geometry of the mouth as a function of time. In order to map the resolved velocity field onto a fixed grid, we interpolated the velocities across the inlet and outlet by average mapping, i.e. we selected a region of interest and averaged the velocity values within this region and set the position at a fixed grid. We calculated the flow rates at the inlet and outlet while changing the averaging regions by integrating the velocity multiplied by (circular strip) the surface across the inlet and outlet for all ages:(4)
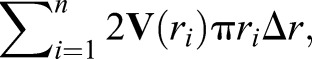


where *n* denotes the total number of circular sections, and **V**(*r*) is the interpolated velocity across the sections. The peak flow rate was flow rate at the time of maximal mouth opening.

To determine the flow conditions within the cavity, we extracted the velocity profile (*U*_mid_) along a vertical line that traverses the center of the second axial length (*L*_2_). Flow along this profile was extracted at the instance where the *L*_2_ has the most parallel orientation (immediately after peak mouth opening). The velocity profiles were calculated at *t*=80.4, 80.4, 77.5, 71.7, 51.5 and 34.4 ms for 8, 13, 18, 23, 30 and 37 dph, respectively.

The Reynolds number (*Re*) was calculated as:(5)
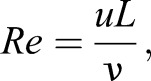
where *u* is flow speed (m s^−1^), ν is the kinematic viscosity of the fluid (m^2^ s^−1^) and *L* is the characteristic length scale (m). We used the swimming speed of the larvae as the characteristic speed and the buccal length as the characteristic length.

The Reynolds number was originally developed to characterize the flow in cases of steady flow within a long rigid tube with a fixed (time independent) radius. However, the suction flow is controlled by the rapid time-dependent motion of the cavity walls, and is characterized by strong temporal flow patterns, which should be taken into consideration. Therefore, we suggest considering the Womersley number, α^2^, which was formulated for pulsating flows mainly associated with cardiovascular systems (Womersley, 1955), and is calculated as:(6)
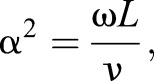
where ω is the characteristic angular frequency (s^−1^), ν is the kinematic viscosity of the fluid (m^2^ s^−1^) and *L* is the characteristic length scale (m). The Womersley number relates the pulsation flow frequency to viscous effects. Here, the angular frequency was calculated using the time it takes the larvae to fully open its mouth; hence, the relevant length scale *L* is gape diameter and the relevant time scale is TTPG, such that ω=2π/TTPG. Using the Womersley number in conjunction with the Reynolds number to characterize suction feeding is advised because it is composed of multiple flow mechanisms that form a coupled interaction within the buccal cavity. Note that we used different (although correlated) characteristic lengths for the calculation of Womersley and Reynolds numbers, referring to buccal cavity length and gape diameter, respectively.

Statistical analysis was performed using the software R statistics (http://www.R-project.org/). ANOVA and linear regression were carried out after verifying homogeneity of variance and normality of residuals. Non-linear fits were fitted using the nls command in the *Stats* library in R.

## RESULTS

**Fig. 3.**
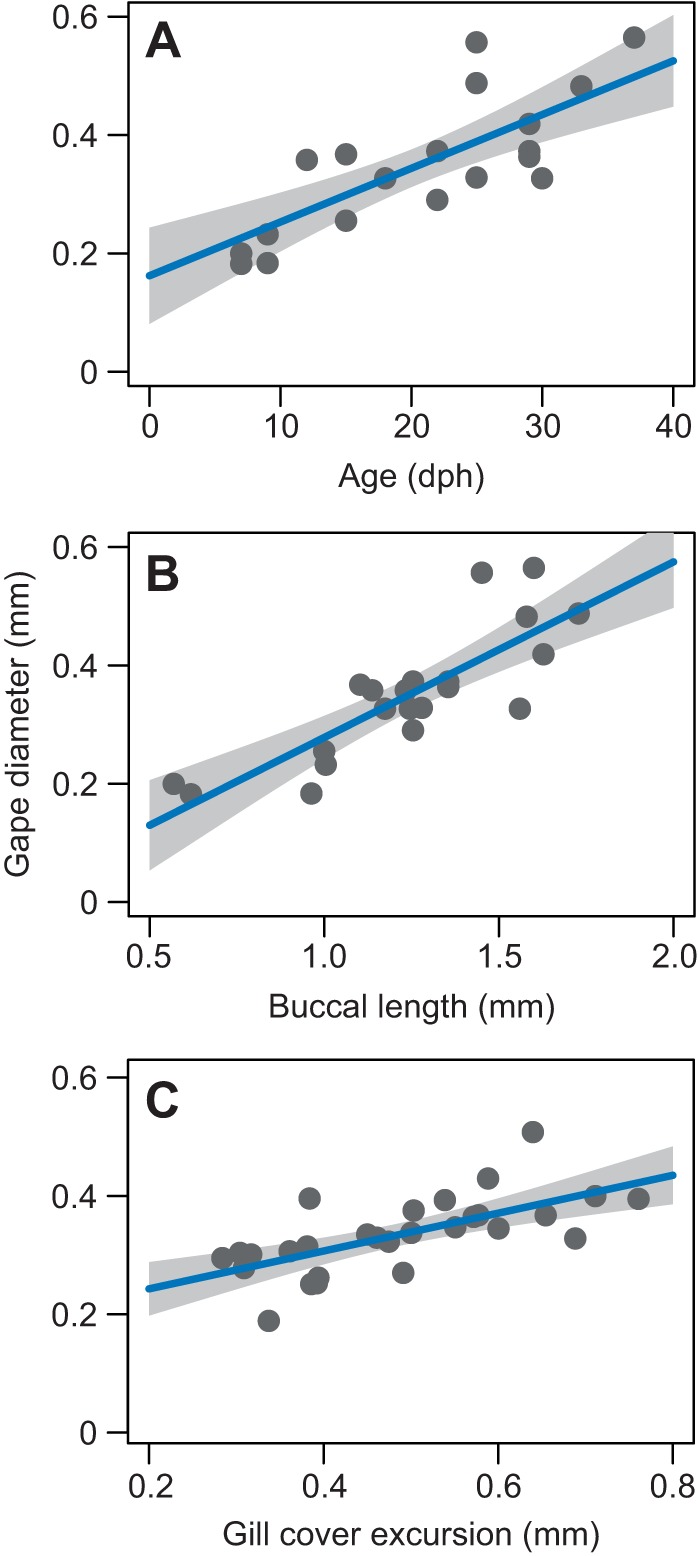
**Gape diameter as a function of age, buccal length and** **gill cover excursion.** The relationship between age (A), buccal length (B) and gill cover excursion (C) and gape diameter in 8–37 dph *S. aurata* larvae (*N*=22 individuals for A and B, and *N*=29 for C). Blue lines depict a linear fit between the two parameters; gray shading depicts standard error of the slope (*R*^2^=0.55, 0.67 and 0.42 for A, B and C, respectively, *P*<0.001 for all).

As larvae matured from 8 dph to 37 dph, the length of the buccal cavity and the diameter of the mouth increased by about twofold. The relationship between age and gape diameter can be described by the equation: peak gape=0.0091(dph)+0.16 (slope s.e.=0.0017; *R*^2^=0.55; *F*_1,20_=27.15, *P*<0.001; Fig. 3A). The relationship between buccal length (*L*) and gape diameter can be described by the equation: peak gape=0.2971(*L*)+0.018 (slope s.e.=0.045; *R*^2^=0.67, *F*_1,19_=42.55, *P*<0.001; Fig. 3B). The excursion of the gill covers also increased with peak gape following the equation: peak gape=0.32(gill excursion)+0.17 (slope s.e.=0.069; *R*^2^=0.42, *F*_1,26_=21.0, *P*<0.001; Fig. 3C). We used the maximal and rest diameters of the gape and gills, and calculated the relationship between approximate gape and gill area, which followed the equation: gill area=0.49(gape area)+0.03 (slope s.e.=0.08; *R*^2^=0.58, *F*_1,26_=37.9, *P*<0.001). Concomitantly, the time to peak gape decreased by a factor of ∼3.6 from an average of 57.3 ms at 8 dph to 15.6 at 37 dph.

**Fig. 4.**
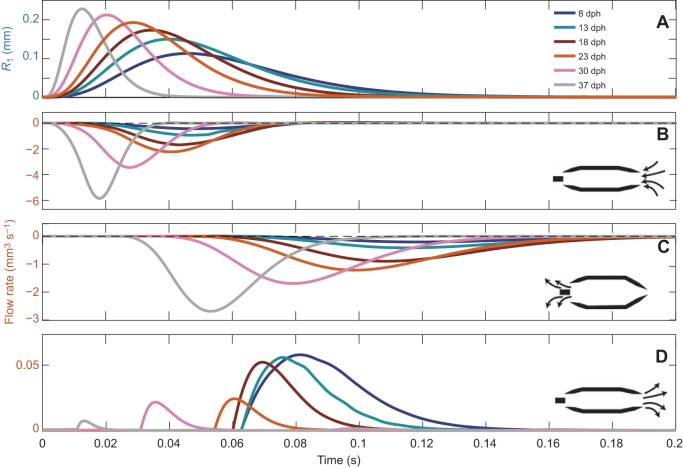
**Mouth size and flow rate as a function of time.** (A) Radius of *R*_1(*t*)_ (gape diameter), (B) influx into the gape, (C) efflux out of the gills and (D) efflux out of the gape. As larvae grow, the influx at the mouth and efflux at the gill increase; however, the efflux at the mouth (flow reversals; positive flow rate) decreases. Note the different scales and units for the *y*-axes in A–D. Negative flow rates indicate flow from right to left (into the mouth and out of the gills).

**Fig. 5.**
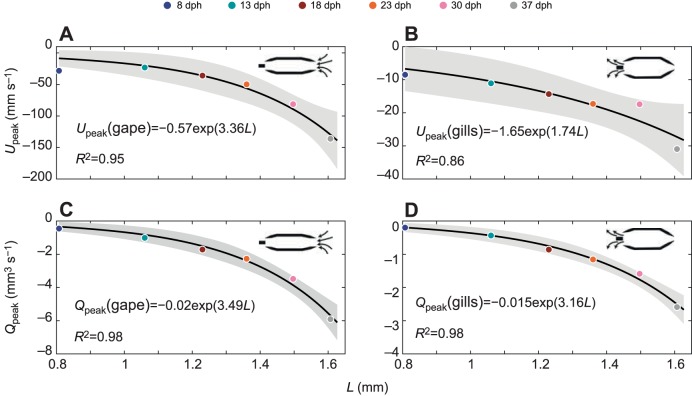
**Scaling of peak flow speed** **(*U*_peak_) and peak flow rate (*Q*_peak_).** Data are shown for gape (A,C) and gills (B,D). *L*, mouth cavity length. Black lines represent exponential fits. Colors depict the different ages. Gray shading represents the confidence interval for the exponential fit. Negative flow speed and rate indicate flow from right to left (into the mouth and out of the gills). See Table S1 for s.e. and details of statistical tests.

By and large, the kinematics observed in *S. aurata* larvae yielded unidirectional flows in our CFD models, i.e. fluid entering the mouth (gape; Fig. 4A,B) and exiting through the gills (Fig. 4C). As previously reported (Yaniv et al., 2014), the magnitude of peak flow velocity at the mouth [*U*_peak_(gape)] increased with increasing buccal length (*L*), and velocity followed the exponential relationship *U*_peak_(gape)=*ae^bL^* where *a*=−0.57 and *b*=3.36 (*R*^2^=0.95; Fig. 5A; *P*-values for *a* and *b* are 0.29 and 0.003, respectively; see Table S1 for s.e. and details of statistical tests). Peak flow speed was 28.3 mm s^−1^ for the 8 dph case and increased to 49.8 and 136.2 mm s^−1^ for the 23 and 37 dph cases, respectively. Correspondingly, *Re* increased by an order of magnitude (from 23 at 8 dph to 218 at 37 dph; Table 1). Similarly, the magnitude of peak flow velocity at the gills [*U*_peak_(gills)] increased with increasing buccal length (*L*), and velocity followed the exponential relationship *U*_peak_(gills)=−1.56*e*^1.74*L*^ (*R*^2^=0.86; Fig. 5B; *P*-values for *a* and *b* are 0.16 and 0.012, respectively; Table S1).

**Fig. 6.**
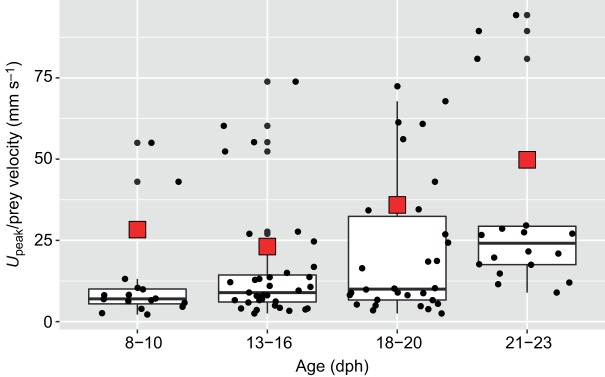
**Model validation.** Comparison of the magnitude of modeled peak flow speed for 8, 13, 18 and 23 dph larvae (this study; red rectangles) and prey velocity measured using a high-speed 3D camera system (China et al., 2017) for 8–23 *S. aurata* larvae (black dots). For prey velocity, black horizontal lines depict the median for each age group, boxes encapsulated the 2nd and 3rd quartiles and whiskers denote 1.5 times the interquartile intervals. Total *N*=100.

Modeled flow speeds for 8, 13, 18 and 23 dph were well within the range of reported peak prey velocities obtained using a 3D high-speed video system (China et al., 2017; Fig. 6). Flow speeds within the cavity were slower near the cavity walls and faster at the center. The velocity profile across the cavity was shallow for 8–23 dph larvae but became steeper for 30 and 37 dph larvae (Fig. S1).

The magnitude of peak flow rate at the mouth [*Q*_peak_(gape)] increased with increasing buccal length from 0.45 mm^3^ s^−1^ for the 8 dph case to 2.27 and 5.91 mm^3^ s^−1^ for the 23 and 37 dph cases, and flow rate followed the exponential relationship *Q*_peak_(gills)=−0.02*e*^3.49*L*^ (*R*^2^=0.98; Fig. 5C; *P*-values for *a* and *b* are 0.1 and 0.001, respectively; Table S1). Peak flow rate at the outlet [*Q*_peak_(gills)] increased with increasing buccal length from 0.20 mm^3^ s^−1^ for the 8 dph case to 1.16 and 2.59 mm^3^ s^−1^ for the 23 and 37 dph cases, and flow rate followed the exponential relationship *Q*_peak_(gills)=−0.015*e*^3.16*L*^ (*R*^2^=0.98; Fig. 5D; *P*-values for *a* and *b* are 0.064 and 0.001, respectively; Table S1).

**Fig. 7.**
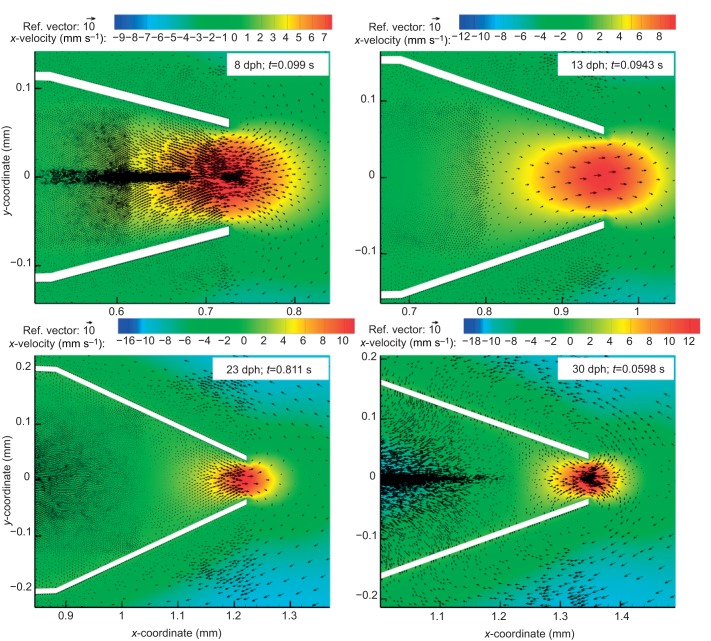
**Vector maps showing peak flow reversal for computational fluid dynamics (CFD) models of 8, 13, 23 and 30 dph larvae.** Vector maps for each age were saved at the time when efflux (flux out of the mouth) was maximal. Different *x*, *y* and speed scale are used in the four panels; however, green consistently represents low (and zero) flows. Also note that gape size at peak efflux decreases with increasing age. Negative flow speeds indicate flow from right to left (into the mouth and out of the gills).

While the observed kinematics in all cases (8–37 dph) resulted in a net influx into the cavity through the gape, we observed considerable efflux (flow reversals) around the time of mouth closure (Fig. 4D, Fig. 7). These flow reversals were most pronounced for models depicting suction feeding in young (8 and 13 dph) larvae, where efflux out of the mouth was ∼11% and ∼4% of the total influx into the cavity, respectively (Fig. 4D, Fig. 7, Table 1). Efflux was negligible (<1% of the influx into the cavity) for 23–37 dph larvae. Influx into the mouth cavity through the gills was negligible (≪0.1% of the influx into the cavity) for all models.

**Fig. 8.**
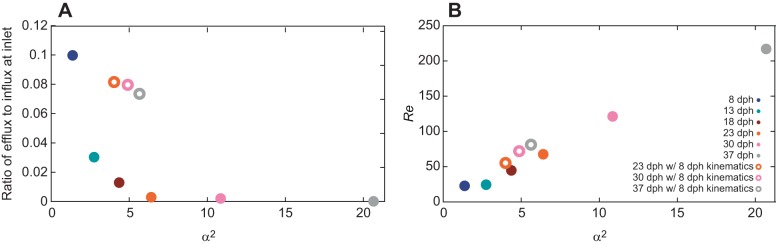
**Hydrodynamic characterization of flow reversals.** (A) Under the original kinematics (filled symbols), the ratio of efflux to influx at the gape decays as the Womersley number (α^2^) decreases, and is most prominent at α^2^<4 and Reynolds number (*Re*)<40 (B). Simulations with larger models where peak excursion and time to peak excursions were similar to the 8 dph case (i.e. representing ‘low-effort’ strikes) were characterized by a high (<8%) ratio of efflux to influx at the gape. These strikes were characterized by *Re*<100 and α^2^<8. While none of the non-dimensional numbers was able to predict the degree of flow reversal, time to peak gape opening (TTPG)>50 ms was related to high backflow.

Plotting the Reynolds versus Womersley numbers for all our cases (Fig. 8) indicated that efflux at the mouth (flow reversals) was >3% of the influx for the smaller larvae, characterized by *Re*<50 and α^2^<4. Furthermore, running the model for the 23, 30 and 37 dph cases using the observed morphology, but with the kinematics of the 8 dph case (‘low-effort’ strikes), yielded high efflux of ∼8–9%, similar to that obtained for the 8 dph case (Table 2, Fig. 8). However, these low effort strikes were characterized by *Re*<100 and α^2^<8, indicating that the backflow phenomena cannot be captured entirely by the two dimensionless numbers.

**Table 2.**
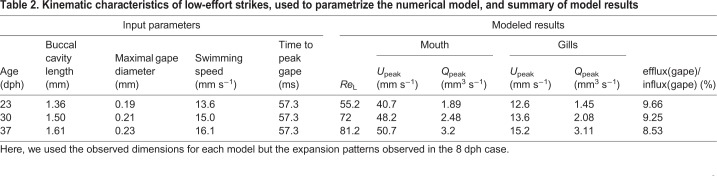
**Kinematic characteristics of low-effort strikes, used to parametrize the numerical model, and summary of model results**

## DISCUSSION

In this study, we used a computational model to investigate the fluid dynamics of suction-feeding larval fish. Using observed strike kinematics of *S. aurata* larvae ranging from first feeding to metamorphosis to parametrize the model, we quantified the flow speeds and the fluxes at the mouth and gills for six larval ages. Throughout larval ontogeny, the dimensions (length and radii) of the buccal cavity increased, and the cavity expanded faster (Figs 2 and 3, Table 1). These kinematics led to an increase in the maximal flow speed and flow rate observed at the gape (Figs 4 and 5). While most of the fluid entering the cavity was expelled through the gills, we observed a high efflux of water flowing outwards from the gape (Figs 4, 7 and 8). These flows occurred predominantly in the models characterized by *Re*<50 and α^2^<4, but also in our larger models during slow mouth opening (low-effort strikes). The low success of larval fish in capturing their prey was previously attributed to insufficient forces exerted by their suction flows on the prey (Yaniv et al., 2014). Here, we demonstrate that reverse flows can develop in small and slow larvae, and we postulate that these flows may carry the prey outside as the mouth closes. This mechanism is expected to be manifested specifically when a small, neutrally buoyant prey does not enter deep enough into the mouth (this study; China et al., 2017; Holzman et al., 2015).

Previous observations of larval feeding on non-evasive prey have indicated the prevalence of in-and-out events in which the prey that entered the mouth was expelled before the mouth closed (China et al., 2017; Holzman et al., 2015). The probability of these in-and-out events increased in suction-feeding events characterized by low *Re* (<20), compared with successful events, which were characterized by a higher *Re* of >40 (China et al., 2017). This observation is in agreement with our present results, indicating the prevalence of high efflux at the gape (flow reversals) under low *Re*, a condition characterizing younger larvae or older larvae that execute low-effort strikes. Furthermore, flow reversal in the models occurred later in the strike, as the mouth was closing. This timing corresponds to the observation of the in-and-out events, and the fact that they were initiated from a greater distance compared with unsuccessful events in which the prey did not enter the mouth at all (China et al., 2017). A flow visualization study in larval zebrafish reported flow reversals that occurred when the mouth starts closing (Pekkan et al., 2016). However, that study did not report the hydrodynamic or kinematic correlates that were associated with the occurrence of flow reversals. For larger fish that operate at higher *Re* [*Re*>55 for 75% of over *N*=400 particle image velocimetry (PIV) measurements; Jacobs and Holzman, 2018], such flow reversals were extremely rare. In general, whether the efficiency of prey transport intra-orally (from the gape towards the esophagus) during suction feeding affects feeding success has rarely been demonstrated.

The Reynolds number is commonly used to characterize the suction flow field for both adult and larval fishes (China and Holzman, 2014; China et al., 2017; Hernández, 2000; Holzman et al., 2015). It provides the ratio between inertia and viscous forces: as the Reynolds number increases, inertial forces are considered dominant over viscous ones, and vice versa. The Reynolds number is frequently used to determine whether the flow is laminar or turbulent (Denny and Wethey, 2001; Vogel, 1994), and for specific cases (e.g. flow within a pipe or around a sphere), critical Reynolds numbers were proposed. However, given the nature of the flow within the buccal cavity, we suggest that the Reynolds number might not convey all the information needed to characterize the fluid phenomena. Unlike the pipe flow for which *Re* was developed, suction flow is a pressure-driven flow, controlled by the rapid time-dependent motion of the cavity walls (Day et al., 2015). Hence, the boundary conditions change as the cavity opens and closes over a short period of time, indicating that suction feeding is not only a pressure-driven phenomenon but also a transient one. Therefore, in addition to the inertia and viscous effects, one should consider the temporal ones. Furthermore, to characterize a suction-feeding event based on the *Re*, one should choose a characteristic length and speed; however, the relevant lengths and speeds are time dependent. For example, peak gape and peak flow speed are commonly used to calculate *Re* for suction-feeding events, but these peak values typically exist for less than a millisecond (Day et al., 2015; van Wassenbergh and Aerts, 2009). As such, the calculated *Re* is a time-dependent number, presumably not suited to characterizing the suction flow regime. We therefore suggest adding, in conjunction with the Reynolds number, the Womersley number (α^2^) which was formulated to assess pulsating flows associated with cardiovascular systems (Womersley, 1955), making this a two scaled parameters problem. Unlike cardiovascular systems, the time between consecutive suction events is relatively long (i.e. the repetition rate is low). However, the characteristic angular frequency (ω, s^−1^) in suction-feeding strikes (i.e. the temporal parameter) is high and therefore dominant within Womersley number. Admittedly, Reynolds and Womersley numbers for different cases can be correlated because their components can be correlated. For example, we use gape diameter to calculate α^2^ and buccal length to calculate *Re*, but the two can be correlated (e.g. Fig. 2B). Additionally, suction flow speed can be correlated with gape size and TTPG (Jacobs and Holzman, 2018). However, we advise that future studies of suction-feeding dynamics report the relevant Womersley number for their case. We re-analyzed data from China et al. (2017) and found that α^2^ was significantly different for failed, successful and in-and-out strikes (ANOVA *F*_2,91_=41.9, *P*<0.0001). Failures were characterized by α^2^=1.01±0.5 (mean±s.d.); in-and-out events were characterized by α^2^=1.31±0.46 and successful strikes by α^2^=2.16±1.33. A *post hoc* analysis revealed that α^2^ was significantly different between successful strikes and failures (*P*<0.001) and between successful strikes and in-and-out events (*P*<0.001), supporting the usefulness of α^2^ for understanding larval feeding.

We hypothesize that the flow reversals stem from the boundary layer that develops near the walls of the mouth, slowing the flow through the mouth. We visualized the flow within the cavity, along a vertical line that traversed the center of the second axial length (*L*_2_), at the instance where *L*_2_ has the most parallel orientation (Fig. S1). The shallow parabolic profile in the cases of 8–23 dph larvae resembled flow in a pipe, suggesting that the flow conditions at the cavity developed from the entrance of two shear layers (axisymmetric boundary layers; BLs) depicted here as two BLs developing inside the mouth. These two BLs, if the mouth cavity was long enough and not moving, would have eventually merged to a uniform flow. However, given the short length of the cavity and the unsteady motion of the boundaries (i.e. closing and opening), we observed a flow that was not fully developed. Regardless, the slope of the flow profile or the estimated width of the boundary layer did not explain the ratio of influx to efflux at the gape, because similar profiles (Fig. S1) were characterized by radically different ratios (Table 1). Essentially, the only variable that could explain flow reversals for the two sets of models (Tables 1 and 2) was TTPG, where TTPG>50 ms was associated with efflux/influx ratios of >1.5%. The logarithm of TTPG was significantly correlated with the degree of flow reversal across all nine models, following the equation: efflux(gape)/influx(gape)=0.12exp^0.12TTPG^ (*R*^2^=0.98; *F*_1,7_=442.9, *P*<0.001). This is consistent with the results of China et al. (2017), in which TTPG of strikes with in-and-out events was the longest of their three categories (success, failure and in-and-out), with a mean (±s.d.) TTPG of 0.04±0.017 s (see their fig. 2F), but the *Re* did not differ significantly between failure and in-and-out events (China et al., 2017).

Our results agree with previous flow visualization studies (Pekkan et al., 2016), modeling (Yaniv et al., 2014), and estimations based on buccal dynamics (China et al., 2017), which reported peak suction flows ranging from 1 to 40 mm s^−1^ for the range of buccal length and gape diameters corresponding to the current study. Additionally, the modeled flow speeds (this study) fell within the range of prey velocity for 8–23 dph *Sparus aurata* larvae (China et al., 2017). Note that these high-speed videos indicate that the variation in peak flow speed among individuals can be substantial (China et al., 2017; Fig. 6). Similarly, PIV studies on adult fish have indicated a broad variation in peak flow speed for repeated strikes by the same individuals (Day et al., 2015; Holzman et al., 2008; Jacobs and Holzman, 2018). Such variation was not factored into our modeling, but could contribute to explaining performance differences between similar-sized larvae (China et al., 2017; Sommerfeld and Holzman, 2019).

## Supplementary Material

Supplementary information
