# Supplementary material for: The hydrodynamic regime drives flow reversals in suction-feeding larval fishes during early ontogeny
Source: J Exp Biol. 2020 Nov 5;223(9):jeb214734. doi: 10.1242/jeb.214734 (PMC7240303; doi:10.1242/jeb.214734)
Supplement: Supplementary information [file jexbio-223-214734-s1.pdf]

Table S1. Details of statistical tests for the curve fitting appearing in Fig. 5.

Figure	Model	Estimate of *a* ±SE	Estimate of *b* ±SE	t-value for *a*	t-value for *b*	P-value for *a*	P-value for *b*
Fig 5A	$U_{peak}(gape) = a\, e^{b\,L}$	-0.57 ±0.47	3.36 ± 0.55	1.2	6.12	0.29	0.003
Fig 5B	$U_{peak}(gills) = a\, e^{b\,L}$	-1.65 ±0.96	1.74 ± 0.39	1.7	4.37	0.16	0.012
Fig 5C	$Q_{inlet}(gape) = a\, e^{b\,L}$	-0.02 ±0.01	3.49 ± 0.30	2.1	11.5	0.1	0.001
Fig 5D	$Q_{outlet}(gills) = a\, e^{b\,L}$	-0.15 ±0.006	3.16 ± 0.25	2.55	12.2	0.064	0.001

Journal of Experimental Biology • Supplementary information

Figure S1.

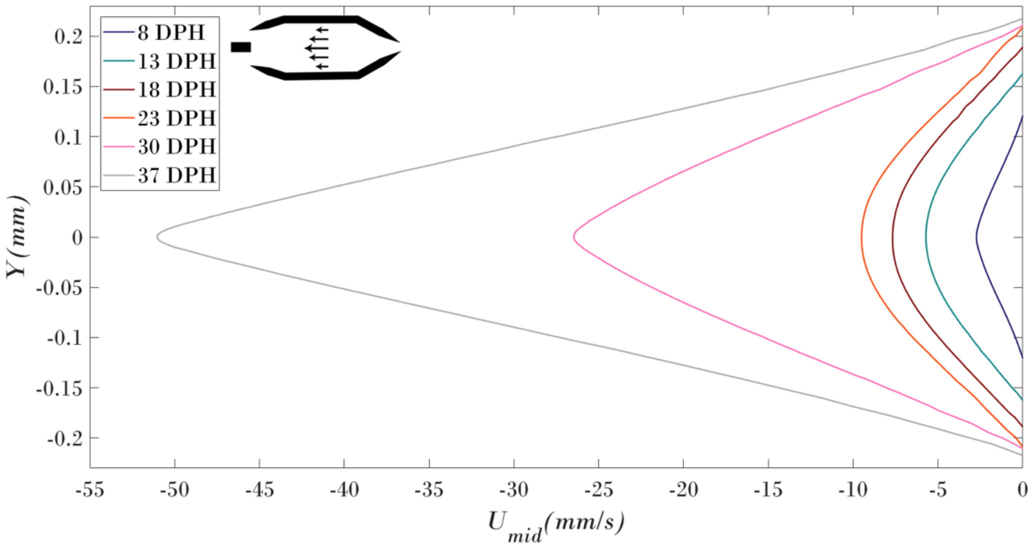

Figure S1: Velocity profiles along a vertical line close to the center of the second axial length (L2) at an instance where the L2 has the most parallel orientation (immediately after peak mouth opening).

Journal of Experimental Biology • Supplementary information
